# First studies on tumor associated carbonic anhydrases IX and XII monoclonal antibodies conjugated to small molecule inhibitors

**DOI:** 10.1080/14756366.2021.2004593

**Published:** 2022-01-20

**Authors:** Chiara Testa, Anna Maria Papini, Reinhard Zeidler, Daniela Vullo, Fabrizio Carta, Claudiu T. Supuran, Paolo Rovero

**Affiliations:** a Interdepartmental Research Unit of Peptide and Protein Chemistry and Biology”, Department of Chemistry “Ugo Schiff”, University of Florence, Sesto Fiorentino, Italy; b Research Group Therapeutic Antibodies, Helmholtz Centre Munich German Research Centre for Environmental Health, Munich, Germany; c Department of Otorhinolaryngology, Klinikum der Universitaet, Munich, Germany; d NEUROFARBA Dept., Sezione di Scienze Farmaceutiche e Nutraceutiche, University of Florence, via Ugo Schff 6, Sesto Fiorentino (Florence), 50019 Italy

**Keywords:** Carbonic anhydrase, monoclonal antibodies, antibody-drug conjugates

## Abstract

We report for the first time Antibody-Drug-Conjugates (ADCs) containing human (h) Carbonic Anhydrase (CA; EC 4.2.1.1) directed Monoclonal Antibodies (MAbs) linked to low molecular weight inhibitors of the same enzymes by means of hydrophilic peptide spacers. In agreement with the incorporated CA directed MAb fragments, *in vitro* inhibition data of the obtained ADCs showed sub-nanomolar K_I_ values for the tumour associated CAs IX and XII which were up to 10-fold more potent when compared to the corresponding unconjugated MAbs. In addition, the introduction of the CA inhibitor (CAI) benzenesulfonamide allowed the ADCs to potently inhibit the housekeeping tumoral off-target human CA II isoform. Such results are supporting the definition of an unprecedented reported class of ADCs able to hit simultaneously multiple hCAs physiologically cooperative in maintaining altered cellular metabolic pathways, and therefore ideal for the treatment of chronic diseases such as cancers and inflammation diseases.

## 1. Introduction

Despite the enormous advances in cancer theranostics, several issues remain unsolved with detrimental consequences on both therapeutic indexes and success rates^1^. New metadata from the World Health Organisation (WHO) on cancers and referred to the last decade, show an increasing numbers of affected patients with a particularly high incidence in 2020 as result of recently established healthcare priorities in response to the ongoing COVID-19 pandemic^2^^,^^3^. Besides the continuous efforts in Medicinal Chemistry to validate novel druggable targets, the panorama in oncology is progressively dominated either by research and clinical studies aimed at improving therapeutic efficacies of established drug, and novel biopharmaceutical drugs such as Antibody–Drug Conjugates (ADCs), which hold great promise as a new class of therapeutics^4^^,^^5^. To date 11 ADCs are marketed for the treatment of hematological and solid tumours, of which six gained regulatory approval since 2019^5^. More importantly larger series are currently facing Phase III investigational stages^5^. From a structural point of view, ADCs are composed of three main components including (i) a monoclonal antibody (MAb) targeting a specific tumour-associated antigen coupled to (ii) a payload (i.e. a cytotoxic drug) by means of (iii) an appropriate linker. Both, the clinical efficacy and the toxicity of ADCs depend on the features of each single component, which together have to ensure the site specific and timely release of the payload, which is often too toxic and/or only has minimal therapeutic activity when administered systemically. The mechanism of action of ADCs is rather complex and strictly depends on each assembled component^6^. It is commonly accepted that a lysosome-based internalisation, upon site specific cellular recognition, is the critical step for ADCs to exert their activity^7^. Nevertheless, ADCs devoid of such feature were also reported to be quite effective *in vivo*, thus offering wider opportunities for cancers treatment as well as in diagnostics^8^^,^^9^. Specifically, non-internalizing ADCs may be ideal when targeting non-internalizing antigens such as the main tumour associated CAs IX and XII, with the result to favour the accumulation of the molecular complex at the tumour site in analogy to recently reported non-internalizing Small Molecule Drug Conjugates (SMDCs) targeting CA IX^10^^,^^11^.

In this context we report on the preliminary investigation of ADCs consisting of CA IX and XII targeting MAbs linked to low molecular weight CA inhibitors (CAIs) by means of a peptide chain.

## 2. Results and discussion

### 2.1. Chemistry

**Scheme 1.**
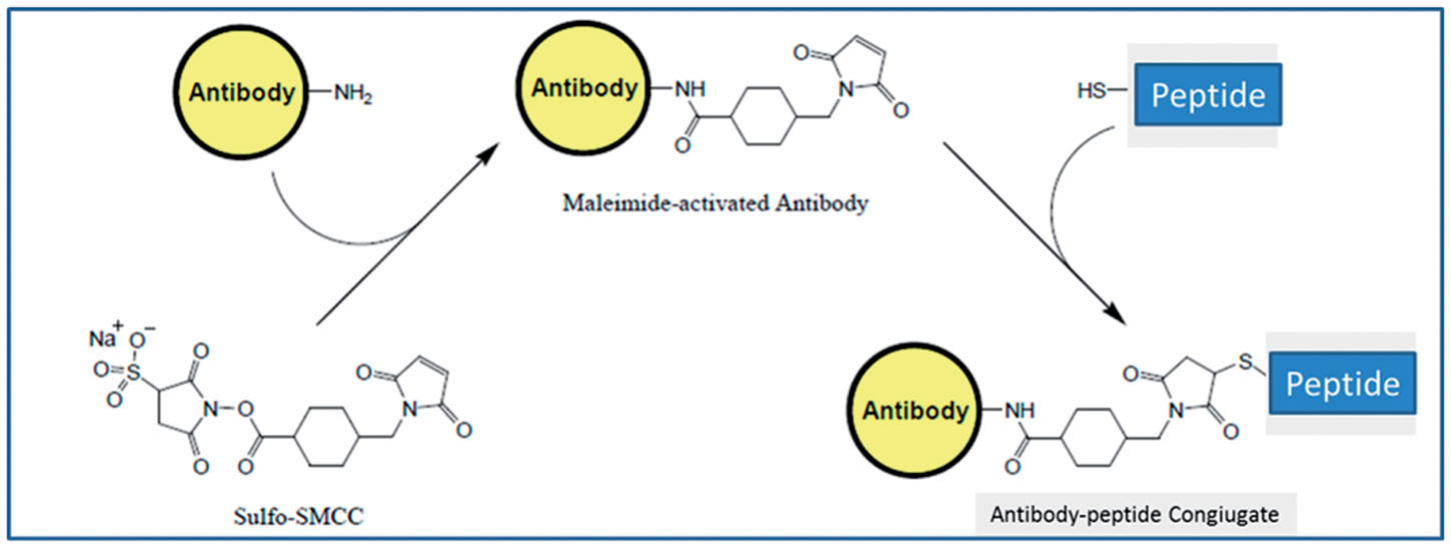
General synthetic procedure for Mab-CA IX/XII conjugates reported in this study.

The mAb-CA IX/XII conjugates reported herein were obtained by means of coupling reactions between either freshly prepared MAb-CA IX and MAb-CA XII antibody solutions with the CAI-containing peptides A and B using the non-cleavable, commercially available bifunctional linker sulfosuccinimidyl 4-(*N*-maleimidomethyl)cyclohexane-1-carboxylate (Sulfo-SMCC) according to Scheme 1.

The coupling procedure was accomplished in two steps: (i) activation of both CAIX- and CAXII-MAb with Sulfo-SMCC used at up to 20-fold molar excess and (ii) conjugation to the terminal cysteine residue of peptides A and B at 1.0 mg peptide/mg antibody ratio. In order to prevent any metal-catalysed sulfhydryl oxidation reactions, the coupling reactions were carried out in the presence of ethylenediaminetetraacetate (EDTA) in PBS buffered aqueous solution. The degree of conjugation was estimated spectrophotometrically using the Ellman’s reagent and measuring the absorbance at 410 nm. This allowed to monitor the progression of the reaction measuring the amount of free sulfhydryl residues from the precursor peptides still present. Usually, all the conjugation reactions resulted complete within 2 h at r.t.

The peptide precursors (A) H-Ala(β-N_3_)-Asp-Lys-Asp-Cys-OH, and (B) H-Pra-Glu-Lys-Glu-Cys-OH were synthesised by solid-phase peptide synthesis following the Fmoc/tBu strategy. The unnatural amino acids containing azido and alkynyl functions (i.e. Fmoc-Ala(β-N_3_)-OH or Fmoc-Pra-OH) were incorporated at the *N*-terminus of the peptide sequences. The insertion of hydrophilic amino acids within the peptide sequences (i.e. Asp, Lys and Glu) was primarily intended either to favour the solubility in aqueous medium of the final products or to promote specificity of interaction with the desired targets^6^.

Solid-phase Cu(I)-catalysed azide-alkyne 1,3-dipolar Huisgen cycloaddition between azido/alkynyl containing peptides A and B with freshly prepared alkynyl/azido containing CAIs 1a–7a^12^^,^^13^ resulted in a series of CAI-functionalized peptides 1–7, which were all characterised by means of RP-HPLC-ESI-MS and resulted ≥95% chromatographic purity (Scheme 2 and Table 1).

**Figure:**
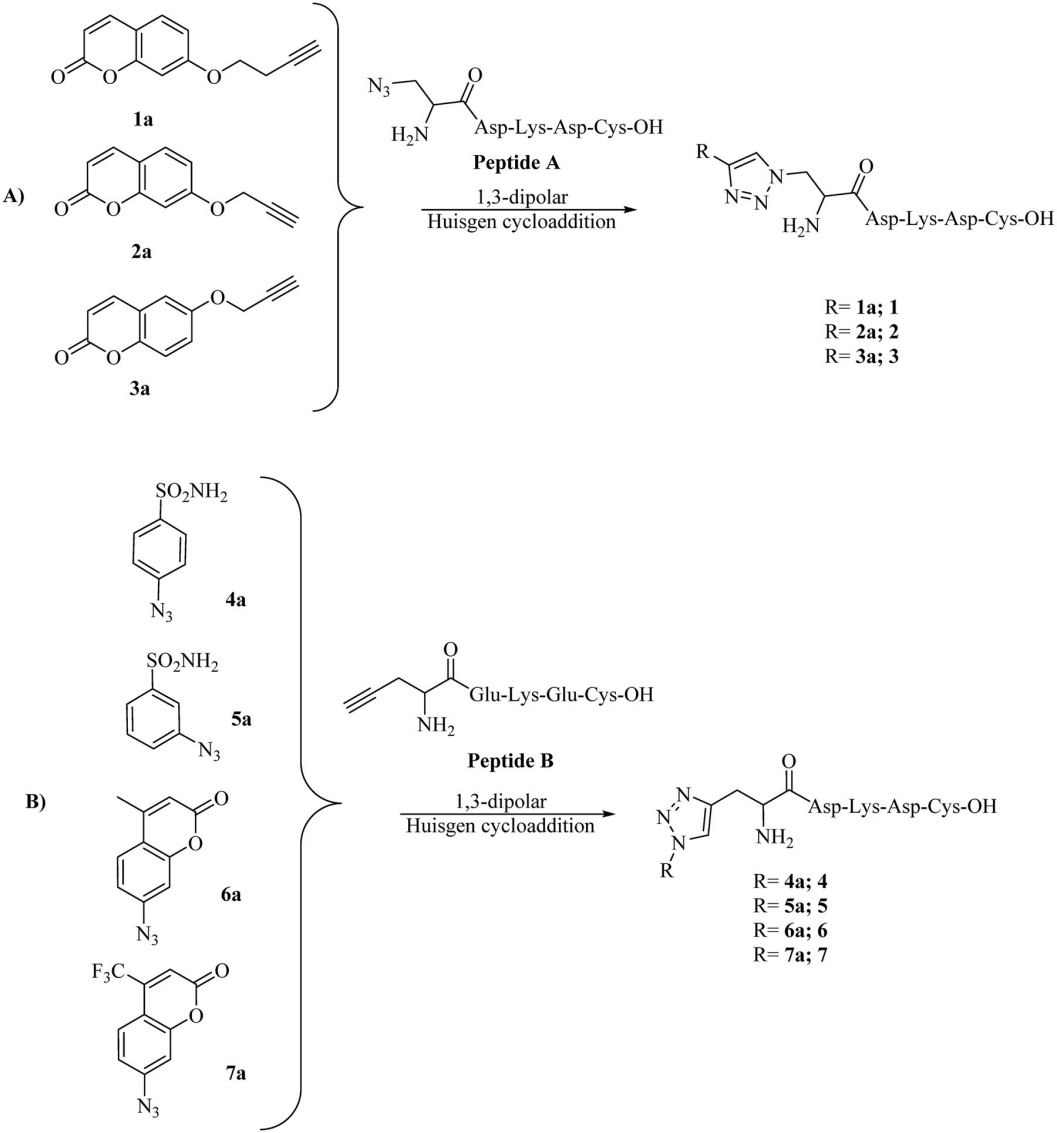
**Scheme 2**. Synthetic approach to CAI-functionalized peptides **1–7**.

**Table 1.** RP-HPLC ESI-MS data for CAI-functionalized peptides 1–7.

Peptides*	Retention time (min)	[M + H]^+^ calculated	[M + H]^+^ found
**1**	13.97	821.30	821.02
**2**	14.04	793.27	792.81
**3**	13.01	793.27	792.81
**4**	11.35	801.27	801.1
**5**	11.37	801.27	801.1
**6**	14.67	803.27	803.04
**7**	16.90	858.27	858.2

*HPLC method: gradient 5%–50% B in 20 min, flow 1 ml/min. A (0.1% TFA in H_2_O) B (0.1% TFA in ACN).

### 2.2. Carbonic anhydrase inhibition

All final ADCs were tested *in vitro* for their inhibitory activity against the abundantly expressed hCAs I, II and the tumour associated hCA IX and XII isoforms in comparison with the reference CAI AAZ (Table 2).

**Table 2.** hCA I, II, IX and XII inhibition data with MAb-CAIX/XII-CAI conjugates using the Acetazolamide (AAZ) as standard by a stopped flow CO_2_ hydrase assay^18^.

Conjugate	K_I_ (nM)*			
	hCA I	hCA II	hCA IX	hCA XII
**1) MAb-CA IX-1**	>50 µM	>50 µM	0.04	>50 µM
**2) MAb-CA IX-2**	>50 µM	>50 µM	0.03	>50 µM
**3) MAb-CA IX-3**	>50 µM	>50 µM	0.06	>50 µM
**4) MAb-CA IX-4**	>50 µM	0.48	0.02	>50 µM
**5) MAb-CA IX-5**	>50 µM	>50 µM	0.05	>50 µM
**6) MAb-CA IX-6**	>50 µM	>50 µM	0.06	>50 µM
**7) MAb-CA IX-7**	>50 µM	>50 µM	0.09	>50 µM
**8) MAb-CA XII-1**	>50 µM	>50 µM	>50 µM	0.70
**9) MAb-CA XII-2**	>50 µM	>50 µM	>50 µM	0.55
**10) MAb-CA XII-3**	>50 µM	>50 µM	>50 µM	0.61
**11) MAb-CA XII-4**	>50 µM	15.1	>50 µM	0.08
**12) MAb-CA XII-5**	>50 µM	>50 µM	>50 µM	0.80
**13) MAb-CA XII-6**	>50 µM	>50 µM	>50 µM	0.79
**14) MAb-CA XII-7**	>50 µM	>50 µM	>50 µM	0.77
**15) MAb-CA IX**	nt	>50 µM	0.3	nt
**16) MAb-CA XII**	>10 µM [15]	>10 µM [15]	640 [15]	3.1[15]
**AAZ**	250	12	25	5.6

*Mean from 3 different assays, by a stopped flow technique^1^ (errors were in the range of ±5%–10% of the reported values). Abbreviations: nt: not tested.

As reported, the MAb-CA IX conjugates (entries 1–7 in Table 2) were ineffective in inhibiting the ubiquitous expressed hCA I as well as the tumour associated hCA XII isoform (K_I_ values >50 µM). As expected, the same conjugates were quite effective in inhibiting the tumour associated hCA IX with K_I_ values at sub-nanomolar concentrations between 0.02 and 0.09 nM (see Table 2). Unfortunately, their kinetic profile on the hCA IX resulted flat and did not allow to properly determine a structure-activity relationship (SAR). It is however of interest that the MAb9 conjugates 1–7 were far more potent in inhibiting hCA IX as compared to the unconjugated MAb-CA IX which itself showed a K_I_ of 0.3 nM (see Table 2). Of particular relevance is the inhibition kinetic profile of the MAb-CA IX conjugates 1–7 on the hCA II isoform, which unexpectedly showed the sulphanilamide conjugate MAb-CA IX-4 (i.e. entry 11) being 25-fold more potent when compared to the reference CAI AAZ (KIs of 0.48 and 12 nM respectively).

As expected, the MAb-CA XII conjugates (i.e. entries 8–14) were ineffective against the hCA I and hCA IX isoforms with K_I_s >50 µM. In contrast, the tumour associated hCA XII was potently inhibited with K_I_s in the sub-nanomolar range (see Table 1). In analogy to the MAb-CA IX conjugates, also in this case the sulphanilamide derivatives, e.g. MAb-CA XII-4, were the only ones active against the hCA II isoform with an inhibition potency comparable to the standard CAI AAZ (K_I_s of 15.1 and 12.0 nM respectively). Interestingly, all Mab-CA XII conjugates were more effective on the hCA XII isoform as compared to the non-conjugated MAb-CA XII which was up to 38.8-fold less potent.

## 3. Conclusions

To the best of our knowledge, this is the first report on the assembly of CA IX and XII directed ADCs loaded with low molecular weight CAIs. This study sets a first line of knowledge on the methodological procedures and analytical set-ups which give access to the development and the evaluation of *ad hoc* designed ADCs and in agreement with required physical/chemical features.

Overall, *in vitro* kinetic inhibition data of the synthesised ADCs on the panel of hCAs considered showed selective and potent inhibition of the tumour associated hCAs IX and XII depending on the MAb, thus proving the reliability of the synthetic methodology pursued. Although the ADC series showed an almost flat kinetic profile on hCAs IX/XII regardless the conjugated CAI, it is interestingly to report they revealed an inhibitory activity that was an order of magnitude higher than that of the corresponding unconjugated MAb. This increased activity is clearly attributable to the contribution of the small molecule CAIs. More importantly, within both the MAb-CA IX and XII ADC series, the benzenesulfonamide moiety was able to induce remarkable inhibition of the hCA II isoform too (i.e. entry 4 and 11 in Table 2). Such results, although unexpected, may be pioneering in defining a new tool able to simultaneously target cooperative CA isoforms involved in sustaining altered cellular metabolisms such as in chronic diseases and cancer, among others.

## 4. Experimental part

### 4.1. Chemistry

Anhydrous solvents and all reagents were purchased from Sigma-Aldrich, Alfa Aesar and TCI. Fmoc-L-Pra-OH was purchased from Iris Biotech GmbH (Marktredwitz, Germany); HBTU was purchased from Advanced Biotech Italy (Milan, Italy); Fmoc-Ala (β-N_3_)-OH was purchased from Sigma-Aldrich. Peptide-synthesis grade N,N-dimethylformamide (DMF) was purchased from Scharlau (Barcelona, Spain); acetonitrile from Carlo Erba (Milano, Italy); dichloromethane (DCM), trifluoroacetic acid (TFA), piperidine, N,N-Diisopropylethylamine (DIPEA), and N-methylmorpholine (NMM) were purchased from Sigma-Aldrich. The scavengers for cleavage of peptides from resin, 1,2-ethanedithiol (EDT), thioanisole, and phenol (PhOH), were purchased from Acros Organics (Geel, Belgium), Jansenn Chimica (Beerse, Belgium), and Carlo Erba (Milano, Italy). All reactions involving air- or moisture-sensitive compounds were performed under a nitrogen atmosphere using dried glassware and syringes techniques to transfer solutions. Nuclear magnetic resonance (^1^H-NMR, ^13^C-NMR) spectra were recorded using a Bruker Avance III 400 MHz spectrometer in DMSO-*d_6_*. Chemical shifts are reported in parts per million (ppm) and the coupling constants (J) are expressed in Hertz (Hz). Splitting patterns are designated as follows: s, singlet; d, doublet; t, triplet; q, quadruplet; m, multiplet; brs, broad singlet; dd, double of doublets. The assignment of exchangeable protons (O*H* and N*H*) was confirmed by the addition of D_2_O. Analytical thin-layer chromatography (TLC) was carried out on Merck silica gel F-254 plates. Flash chromatography purifications were performed on Merck Silica gel 60 (230–400 mesh ASTM) as the stationary phase and ethyl acetate/*n*-hexane were used as eluents. Melting points (mp) were measured in open capillary tubes with a Gallenkamp MPD350.BM3.5 apparatus and are uncorrected. The lyophilised crude peptides were initially treated by solid-phase extraction with a RP-18 LiChroprep silica column from Merck (Darmstadt, Germany) using H_2_O/ACN as eluent yielding a partially purified product. The final purification of the partially pure peptides was performed by semi-preparative RP-HPLC on a Phenomenex Jupiter C-18 (250 mm 34.6 mm) column at 288 °C using a Waters instrument (separation module 2695, detector diode array 2996) working at a flow rate of 4 ml/min. The solvent system used was: A (0.1% TFA in H_2_O, v/v) and B (0.1% TFA in 84% CH_3_CN in A, v/v). The solvent gradient was 0.5%–50% B in 20 min. Final purity of all peptides was 95%. Peptides were characterised by RP-HPLC ESI-MS. Analytical HPLC system was an Alliance Chromatograph (Waters) with a Phenomenex Kinetex C-18 column 2.6 µ (100 mm x 3.0 mm) working at a flow rate of 0.6 ml/min, with UV detection at 215 nm, coupled to a single quadrupole ESI-MS (Micromass ZQ). The solvent systems used were: A (0.1% TFA in H_2_O, v/v) and B (0.1% TFA in 84% CH_3_CN in A, v/v).

### 4.2. Solid-phase peptide synthesis

The peptide precursors A and B were synthesised on Fmoc-Cys(Trt)-Wang resin (0.57 mmol/g, 500 mg), on a manual batch synthesiser (PLS 4 × 4, Advanced ChemTech), following the Fmoc/tBu chemistry. The resin was swelled with DMF (1 ml/100 mg of resin) for 20 min before use. Stepwise peptide assembly was performed by repeating deprotection-coupling cycles with the required amino acids. In brief: (a) Swelling: DMF (1 ml/100 mg of resin) for 5 min. (b) Fmoc-deprotection: resin washing with 20% (v/v) piperidine in DMF (1 ml/100 mg of resin, one wash for 5 min, followed by another wash for 20 min). (c) Resin washing: DMF (3–5 min). (d) Coupling: HBTU/NMM (5.0/7.0 equiv.) as coupling system and 5 eq. of the Fmoc-protected amino acids, except for the non-coded amino acids Fmoc-L-Ala(β-N_3_)-OH and Na-Fmoc-L-Pra-OH, for which 2.5 eq. were used. The coupling was carried out in DMF (1 ml/100 mg of resin) for 50 min. (e) Resin washings: DMF (3–5 min) and DCM (1–5 min). Each coupling was monitored by Kaiser test and was negative at completion, therefore recouplings were not needed. The resin-bound peptide was subjected to solid-phase Cu(I)-catalysed azide-alkyne 1,3-dipolar Huisgen cycloaddition (CuAAC). To the dry resin bound peptide in a fritted syringe were added CuI (1.0 eq), sodium ascorbate dissolved in water (1.0 eq.), the appropriate CAI-alkynyl 1a–3a or CAI-azide 4a–7a section (1.0 eq.), DIPEA (10.0 eq.), and 2,6-lutidine (10.0 eq.) in 1 ml DMF. After 18 h at r.t. the resin was filtered and washed with DMF and DCM. Peptide cleavage from the resin was carried out by shaking the peptidyl resin for 3 h at room temperature in a mixture of TFA/anisole/1,2-ethanedithiol/phenol/H_2_O (94:1:1:1:1, v/v/v/v/v, 1 ml/100 mg of resin-bound peptide). This led also to the deprotection of the amino acid side chains. Resin was filtered and washed with TFA. The crude peptide was recovered by centrifugation after concentration of the filtrate under N_2_ stream and precipitation by addition of cold diethyl ether. The pellet was dissolved in H_2_O and freeze-dried. The lyophilised crude peptides were partially purified by solid-phase extraction and then purified by semipreparative RP-HPLC with a linear solvent gradient of 0.5%–50% B in A in 20 min. The final chromatographic purity of all peptides was ≥95%. Peptides were characterised by RP-HPLC-ESI-MS.

### 4.3. Conjugation

The MAb-CAI conjugates were obtained by means of reaction couplings, using the commercially available bifunctional linker Sulfo-SMCC, between the MAb-CAIX and MAb-CAXII antibodies with the CAI-containing peptides A and B. 100 µL of the appropriate antibodies in PBS buffer were added to a solution of the bifunctional linker Sulfo-SMCC (20-fold molar excess) in conjugation buffer (0.1 M sodium phosphate, 0.15 M NaCl, pH 7.2). The reaction mixture was incubated 1 h at room temperature. Then the excess of crosslinker was removed using a desalting column equilibrated with the conjugation buffer. After the antibody has been activated with Sulfo-SMCC, the degree of maleimide incorporation was detected by Ellman’s test. The cysteine containing peptide (CAI-containing peptides A and B) were dissolved in the conjugation buffer (0.1 M sodium phosphate, 0.15 M NaCl, 0.1 M EDTA pH 7.2) and added to the activated antibody using 1 mg peptide/mg antibody. EDTA was used to prevent metal-catalysed sulfhydryl oxidation to disulphides. The conjugation reaction was carried out for 2 h at room temperature. The degree of conjugation was estimated by assaying the amount of sulfhydryl present before and after the coupling reaction, using Ellman’s reagent. The absorbance (410 nm) of the peptide and of the activated protein before the conjugation was compared to the absorbance of the reaction mixture

### 4.4. Mab Antibodies

The monoclonal CA IX-specific antibody was generated by immunising rats with extracellular vesicles derived from RBL-1 cells (ATCC CRL-1378) overexpressing human CAIX. Hybridoma were generated and the specificities of the secreted antibodies was tested by flow cytometry against parental HEK293 cells (which are CAIX-negative) and a subclone overexpressing human CAIX. Generation of the CAXII-specific antibody '6A10' has been described elsewhere^14^.

### 4.5. Carbonic anhydrase inhibition

An Applied Photophysics stopped-flow instrument has been used for assaying the CA catalysed CO_2_ hydration activity^14^. Phenol red (at a concentration of 0.2 mM) has been used as indicator, working at the absorbance maximum of 557 nm, with 20 mM Hepes (pH 7.5) as buffer, and 20 mM Na_2_SO_4_ (for maintaining constant the ionic strength), following the initial rates of the CA-catalysed CO_2_ hydration reaction for a period of 10–100 s. The CO_2_ concentrations ranged from 1.7 to 17 mM for the determination of the kinetic parameters and inhibition constants. For each inhibitor at least six traces of the initial 5%–10% of the reaction have been used for determining the initial velocity. The uncatalyzed rates were determined in the same manner and subtracted from the total observed rates. Stock solutions of inhibitor (0.1 mM) were prepared in distilled-deionized water and dilutions up to 0.01 nM were done thereafter with the assay buffer. Inhibitor and enzyme solutions were preincubated together for 15 min at room temperature prior to assay, in order to allow for the formation of the E-I complex. The inhibition constants were obtained by non-linear least-squares methods using PRISM 3 and the Cheng–Prusoff equation, as reported earlier^15–17^ and represent the mean from at least three different determinations. All CA isoforms were recombinant ones obtained in-house as reported earlier^15–17^.
